# Acyl protein thioesterase 1 and 2 (APT-1, APT-2) inhibitors palmostatin B, ML348 and ML349 have different effects on NRAS mutant melanoma cells

**DOI:** 10.18632/oncotarget.6907

**Published:** 2016-01-13

**Authors:** Igor Vujic, Martina Sanlorenzo, Rosaura Esteve-Puig, Marin Vujic, Andrew Kwong, Aaron Tsumura, Ryan Murphy, Adrian Moy, Christian Posch, Babak Monshi, Klemens Rappersberger, Susana Ortiz-Urda

**Affiliations:** ^1^ Department of Dermatology, Mt. Zion Cancer Research Center, University of California San Francisco, San Francisco, CA, USA; ^2^ Department of Dermatology, The Rudolfstiftung Hospital, Academic Teaching Hospital, Medical University Vienna, Vienna, Austria; ^3^ Department of Medical Sciences, Section of Dermatology, University of Turin, Turin, Italy; ^4^ Department of Dermatology, Brigham and Women's Hospital, Harvard Medical School, Boston, MA, USA

**Keywords:** NRAS, APT-1, melanoma, palmostatin B, acyl protein thioesterase

## Abstract

Oncogenic NRAS mutations are frequent in melanoma and lead to increased downstream signaling and uncontrolled cell proliferation. Since the direct inhibition of NRAS is not possible yet, modulators of NRAS posttranslational modifications have become an area of interest. Specifically, interfering with NRAS posttranslational palmitoylation/depalmitoylation cycle could disturb proper NRAS localization, and therefore decrease cell proliferation and downstream signaling. Here, we investigate the expression and function of NRAS depalmitoylating acyl protein thioesterases 1 and 2 (APT-1, APT-2) in a panel of NRAS mutant melanoma cells. First, we show that all melanoma cell lines examined express APT-1 and APT-2. Next, we show that siRNA mediated APT-1 and APT-2 knock down and that the specific APT-1 and -2 inhibitors ML348 and ML349 have no biologically significant effects in NRAS mutant melanoma cells. Finally, we test the dual APT-1 and APT-2 inhibitor palmostatin B and conclude that palmostatin B has effects on NRAS downstream signaling and cell viability in NRAS mutant melanoma cells, offering an interesting starting point for future studies.

## INTRODUCTION

Cutaneous melanoma is an aggressive skin cancer with rising incidence [1]. Early stage melanomas can be cured by surgical excision, but the metastatic disease still has a very poor outcome.

In melanoma, driving oncogenic mutations in v-Raf murine sarcoma viral oncogene homolog B (BRAF) and neuroblastoma rat sarcoma viral oncogene homolog (NRAS) are found in about 40% and 20% of tumors, respectively [2–4]. These mutations lead to excessive downstream signaling, increased cell growth and uncontrolled proliferation [5–7]. BRAF inhibitors vemurafenib and dabrafenib, selectively block the mutant BRAF protein and prolong overall survival in patients with BRAF mutant melanoma [8,9]. On the other hand, attempts to directly block NRAS have not been successful so far and patients with these mutations have a worse prognosis [10]. The inhibition of components of NRAS's downstream cascades has shown promising data in vitro but inconsistent results in patients [11–17]. Oncogenic NRAS mutations occur not only in melanoma, but also in a variety of other cancers, which further emphasizes the need for studies on substances that interfere with NRAS signaling [18,19].

NRAS and its close homolog, Harvey rat sarcoma viral oncogene homolog (HRAS), belong to the family of small GTPases. Under normal conditions, the proteins connect membrane-bound receptor tyrosine kinases (RTKs) to intracellular signaling networks and are critical regulators of cell fate and cell cycle progression. Oncogenic mutations lock the proteins in their active, GTP bound state and cause continuous downstream signaling, promoting cell division and tumor growth [6,20,21]. Interestingly, NRAS and HRAS signaling, depends upon their intracellular localization; therefore, interfering with this localization has the potential of modulating RAS activity [22–24].

The proper NRAS and HRAS plasma membrane localization is regulated by the addition of palmitic acids to the C-terminus of the proteins (palmitoylation). The palmitoylation occurs in the Golgi apparatus and the palmitoylated proteins travel to the plasma membrane where they bind their signaling partners. On the plasma membrane the proteins are then depalmitoylated and recycle back to the Golgi apparatus. This dynamic cycle is tightly regulated by palmitoyl transferases (palmitoyaltion) and acyl protein thioesterases 1 and 2 (APT-1, APT-2) (depalmitoylation) [24,25]. Consequently, these enzymes are potential therapeutic targets in cells with hyperactive NRAS or HRAS signaling. The recently developed APT-1 and -2 inhibitor palmostatin B selectively inhibited the growth of NRAS^G12D^ mutant hematopoietic cells and HRAS^G12V^ transformed fibroblasts by disturbing NRAS and HRAS localization to down-regulate RAS signaling [26,27]. Based on these encouraging results we hypothesized that interference with APT-1 and APT-2 function might be beneficial in NRAS mutant melanoma, for which FDA approved targeted therapies are still lacking.

Here, we investigate APT-1 and APT-2 expression and function in a panel of NRAS mutant melanoma cell lines. We show that siRNA mediated knockdown or their inhibition with highly specific inhibitors ML348 and ML349 does not down-regulate NRAS signaling or decrease cell viability. In contrast, palmostatin B showed a dose-dependent reduction of cell viability in a panel of NRAS mutant melanoma cell lines.

## RESULTS

### Acyl protein thioesterases APT-1 and APT-2 are expressed in melanoma cell lines

**Figure 1:**
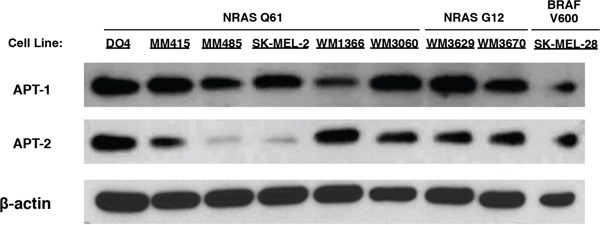
Immunoblots of APT-1 and APT-2 expression in melanoma cell lines Melanoma cell lines with activating NRAS mutations in exons II (NRAS G12) and III (NRAS Q61), and the BRAF V600 mutant SK-MEL-28 express APT-1 and APT-2 protein.

Inhibition of acyl protein thioesterases 1 and 2 (APT-1, APT-2) interferes with NRAS localization and cell growth in NRAS mutant hematopoietic cells, but the function of the proteins in NRAS mutant melanoma is unknown [27]. To elucidate the role of the two close homologs APT-1 and APT-2, we selected a panel of NRAS mutant melanoma cell lines based on their previously characterized NRAS mutations in exons II and III and compared them to the BRAF mutant line SK-MEL-28, which also hyper-activates the MAPK pathway through BRAF^V600E^ but not through mutant NRAS [12,13,28,29]. First, we analyzed APT-1 and APT-2 protein expression. Both proteins were ubiquitously expressed in all cell lines examined, albeit at different levels (Figure 1).

### APT-1 and APT-2 knock down in NRAS mutant melanoma does not alter cell viability or NRAS downstream signaling

**Figure 2:**
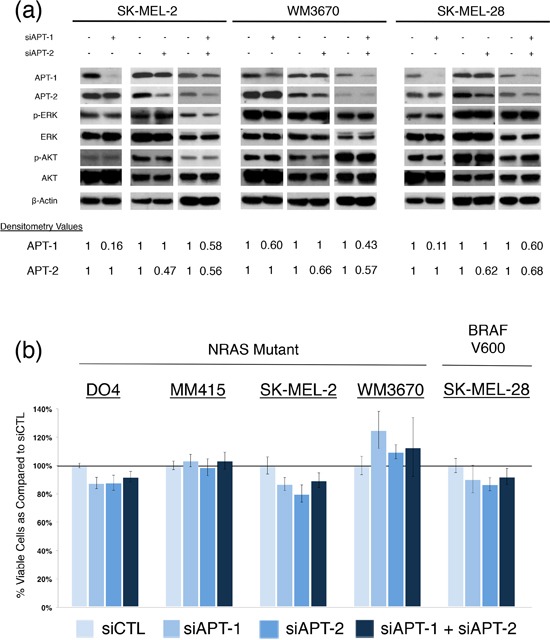
siRNA knock down of APT-1 and APT-2 in NRAS and BRAF mutant melanoma **(a)** and **(b)** melanoma cells with indicated mutations were transfected with APT-1 or APT-2 targeting siRNA pools (siAPT1, siAPT2) or their combination and compared to cells transfected with a non-targeting siRNA pool (concentration 100nM, incubation 72hrs). **a.** Immunoblots after APT-1, APT-2 or dual knock down. Densitometry values for APT-1 and APT-2 show the percentage of protein expression following siRNA treatment. No relevant changes in NRAS downstream effectors are seen following APT-1, APT-2 or dual knock down. **b.** Bar graphs depicting viable cells after APT-1, APT-2 or dual knock down. Analyses revealed no significant differences in cell viability in NRAS and BRAF mutant melanoma cells (incubation 72hrs, *n* > 3, error bars represent SD)

**Figure 3:**
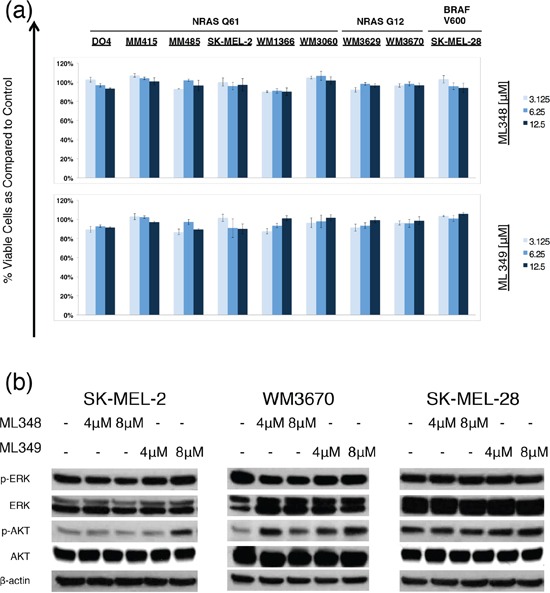
Effects of APT-1 and APT-2 inhibitors ML348 and ML349 effects on melanoma cells **a.** Dose response bar graphs of melanoma cells with NRAS mutations in exon II (NRAS G12), exon III (NRAS Q61), or with BRAF mutation (BRAF V600) treated with the APT-1 inhibitor ML348 or APT-2 inhibitor ML349 compared to DMSO treated controls (incubation 72hrs, *n* = 3, error bars represent SD). ML348 and ML349 do not decrease cell viability in melanoma cells at dosages used in this study. **b.** Immunoblot analyses for NRAS downstream effector proteins (incubation 6hrs). Analyses show slight changes of AKT phosphorylation in NRAS mutant cells SK-MEL-2 and WM3670.

Since all cell lines expressed both proteins and their inhibition has the potential to suppress mutant NRAS function in melanoma, we utilized siRNA to knock down APT-1 and APT-2. Because both homologs (65% sequence identity) can depalmitoylate NRAS, we also studied the dual knock down of APT-1 and 2 [25]. We confirmed and quantified the protein knock down, and evaluated the main NRAS downstream cascades, which are important for survival and growth in NRAS mutant cancer [11,30,31]. After APT-1 and APT-2 knock down the phosphorylation of NRAS down streamers ERK and AKT remained unchanged in NRAS mutant melanoma cells (Figure 2). In line with this, partial siRNA mediated knock down of APT-1, APT-2, or both did not affect cell viability in melanoma cell lines tested (Figure 3).

### Specific APT-1 and APT-2 inhibitors ML348 and ML349 do not affect cell viability in NRAS mutant melanoma cells

Transient siRNA mediated APT-1 and APT-2 knockdown was effective, but did not completely abolish APT-1 and APT-2 protein levels. Thus, we evaluated recently synthesized compounds ML348 and ML349. Which are potent APT-1 and APT-2 inhibitors designed to study APT-1 and -2 functions and might lead to a better substrate inhibition than achieved with siRNAs. Both drugs are highly substrate specific and did not have any cytotoxic effects on human embryonic kidney cells (HEK293T) [32,33]. We used the maximum soluble drug concentrations in supplemented cell growth media at room temperature (<12.5 μM) [32,33]. ML348 and ML349 did not decrease cell viability, but they led to a slight activation of AKT in NRAS mutant cells, while no such effect was seen in the BRAF mutant SK-MEL-28 (Figure 3). No effects were observed on the main NRAS effector p-ERK.

### The APT-1 and APT-2 inhibitor palmostatin B decreases cell viability in NRAS mutant melanoma cell lines

**Figure 4:**
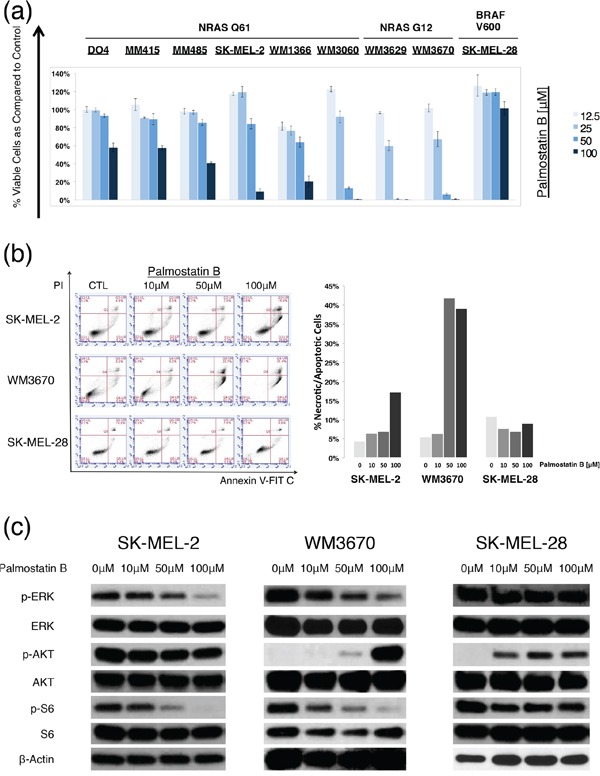
Palmostatin B effects on NRAS mutant melanoma cells **a.** Dose response bar graphs of melanoma cells with NRAS mutations in exon II (NRAS G12), exon III (NRAS Q61), or BRAF mutations (BRAF V600) treated with the APT-1 and -2 inhibitor palmostatin B compared to DMSO treated controls. Palmostatin B shows a dose-dependent effect on cell viability in all NRAS mutant melanoma cell lines, but not in the BRAF mutant control (incubation 72hrs, n=3, error bars represent SD). **b.** Representative flow cytometry dot blots from cells treated with palmostatin B. Palmostatin B leads to a dose dependent increase of cell death (upper right quadrant) in NRAS mutant, but not in BRAF mutant melanoma cell lines. Bars represent the relative number of apoptotic/necrotic cells compared to DMSO treated controls (*t* = 48hrs). **c.** Immunoblot analyses for main NRAS downstream effectors after treatment with palmostatin B (incubation 6hrs). Palmostatin B shows a dose-dependent down-regulation of ERK and S6 phosphorylation in NRAS mutant but not in BRAF mutant melanoma cells.

Palmostatin B is another recently developed APT inhibitor. In previous studies it selectively decreased cell growth in NRAS mutant, but not in KRAS mutant or wild type cells in dosages of up to 100 μM. Palmostatin B primarily inhibits APT-1 and APT-2, but may have off target effects on other serine hydrolases [25–27,32]. We tested the drug on our melanoma cell panel and at dosages similar to previous reports. In contrast to ML348 and ML349, palmostatin B led to a dose dependent cell viability decrease in most NRAS mutant cell lines, while no significant cell viability decrease was observed in the BRAF mutant cell line SK-MEL-28 (Figure 4). The GI50 values (concentrations of drugs resulting in 50% decrease in cell viability relative to DMSO treated controls) ranged from 9.93 μM for the cell line WM3670 to >100 μM for MM415 and the BRAF mutant SK-MEL-28 (Supplementary Table S1).

Next, we selected cell lines that had significant decreases in cell viability after palmostatin B incubation and studied the induction of apoptosis or necrosis via Annexin V/Propidium Iodide staining followed by flow cytometry. The apoptosis assays were in line with the CellTiter-Glo (CTG) assays used for the dose response curves, and revealed that palmostatin B leads to dose-dependent cell death in NRAS mutant cell lines WM3670 and SK-MEL-2, but not in the BRAF mutant cell line SK-MEL-28 (Figure 4). In contrast to ML348 and ML349, palmostatin B caused a dose-dependent decrease in phosphorylation of the main NRAS downstream effectors ERK and S6 (Figure 4). The induction of AKT phosphorylation in WM3670 and Sk-MEL-28 by APT inhibition with palmostatin B can be explained by the decrease of the negative feedback mechanisms exerted through less active ERK in some cell lines [34–36].

## DISCUSSION

RAS proteins are important regulators of cell fate and up to 30% of all cancers have driving mutations in KRAS, HRAS or NRAS [20]. So far, efforts to design and deliver specific inhibitors of mutant RAS *in vivo* have failed and new approaches to treat RAS mutant cancer are needed. Targeting the post-translational modification of RAS mutant melanoma is not a novelty: more than a decade ago *in vitro* and *in vivo* mouse studies with farnesyltransferase inhibitors showed potent target inhibition with little associated cytotoxicity [37,38]. Yet, phase II clinical trials had to be aborted because none of the enrolled patients showed clinical response[39].

Recent reports show that the pharmacological interference of the dynamic HRAS and NRAS palmitoylation/depalmitoylation cycle through inhibition of APT-1 and -2 selectively reduces growth and signaling in cells with oncogenic HRAS or NRAS mutations [26,27]. Here, we test if this also applies to NRAS mutant melanoma, where such mutations are found in 20% of tumors and treatment options are limited [40].

The NRAS depalmitoylating enzymes APT-1 and 2 could be potential targets in NRAS mutant melanoma as they regulate the subcellular localization of NRAS, which in turn affects its downstream signaling. Our study shows that all tested melanoma cell lines express both proteins, albeit at different levels (Figure 1). These results are consistent with published mRNA expression data, where all 61 melanoma cell lines tested expressed mRNA for both proteins, underlining their importance for cell survival [28]. To our knowledge there are no reports on APT-1 and -2 functions in NRAS mutant melanoma.

To examine the biological function of APT-1 and -2 in NRAS dependent cell growth and signaling, we knocked down both proteins using siRNA. For our experiments we chose a panel of melanoma cells with activating NRAS mutations in codons 12 and 61, which we compared to a BRAF^V600^ mutant and NRAS wild type melanoma cell line. To our surprise, siRNA mediated APT-1, APT-2, or dual knock down did not decrease cell viability or affect the major NRAS downstream effectors (Figure 2).

As the siRNA did not completely abolish APT-1 and 2 we used newly synthesized APT-1 and 2 inhibitors ML348 and ML349, which have very strong substrate inhibition. These compounds were specifically designed for studying these proteins and their specificity and *in vivo* APT-1 and 2 inhibition has been confirmed in previous studies [32,33]. ML348 and ML349 did not cause any decrease in cell viability or consistent changes in the main NRAS down streamers ERK and AKT. Though higher concentrations of the two compounds might affect cell biology, the use of ML348 and ML349 in supplemented media is limited by drug solubility. On the other hand, both compounds showed very high and selective bioactivity scores at 5 μM in HEK293T cells and we expect that similar substrate inhibition is achieved in melanoma cells [41]. ML348 and ML349's lack of significant effects are in line with our siRNA studies and suggest a negligible effect of APT-1 and 2 inhibition in NRAS mutant melanoma growth and downstream signaling.

To further investigate roles of APT-1 and -2 in NRAS mutant melanoma, we tested another newly developed drug, palmostatin B. Its different chemical structure may render it less specific, compared to ML349 and ML349 [32,42]. However, it is the only APT inhibitor that has shown effects on cell viability and RAS downstream signaling in HRAS and NRAS mutant cells [26]. The drug resulted in a dose-dependent cell viability decrease in all NRAS mutant cell lines tested. Interestingly, the effect was significantly more pronounced in cells with NRAS mutations in exon II (codon G12) than in exon III (codon Q61) (*p* <.05, Mann-Whitney-U test), where the GI50 values were lower and comparable to previous reports in other NRAS^G12^ mutant cells [26,42]. This finding might be explained by the fact that NRAS^Q61^ mutant proteins have decreased GTPase activity and increased stability compared to NRAS^G12^ mutants, leading to more active, GTP-bound protein in cells with NRAS^Q61^ mutations [43]. It is tempting to speculate whether cells with mutations in NRAS^G12^ might be more prone to interference of NRAS localization, as the overall downstream signaling is weaker. In agreement with the cell viability decrease we observed a dose dependent reduction of main NRAS downstream signaling effectors p-ERK and p-S6 (Figure 4). Our results are supported by previous studies which show that palmostatin B mediated inhibition of APT-1 and -2 affects HRAS^G12^ transformed fibroblasts, NRAS^G12^ transformed fetal liver cells and NRAS mutant leukemia cells [26,42]. To our knowledge this is the first report of APT-1 and APT-2 inhibitor activity in NRAS mutant melanoma cells. Still, before we can estimate palmostatin Bs' real potential in the treatment of NRAS mutant melanoma, further *in vivo* studies are needed and warranted. As there are no data so far about the pharmacokinetics of palmostatin B, first its oral and intravenous bioavailability should be measured and serum drug concentrations should be quantified. After finding the right dose regimen and its lethal dose in mice, melanoma xenograft models would offer a good platform for first tests of palmostatin B *in vivo* action.

In summary, we did not observe biologically significant effects on cell growth and NRAS signaling following specific APT-1 and/or APT-2 inhibition in a panel of NRAS mutant melanoma cell lines using siRNA and the specific inhibitors ML348 and ML349. Palmostatin B, however, decreased NRAS downstream signaling and cell viability, but the moderate micro molar dosages may have off-target effects. So far no *in vivo* studies have been performed but the first palmostatin B results in NRAS mutant melanoma are promising and warrant further studies evaluating if and to what extent palmostatin B interferes with NRAS localization in NRAS mutant melanoma.

## MATERIALS AND METHODS

### Cell lines, cell culture

Human NRAS mutated melanoma cell lines DO4, MM415, MM485, SK-MEL-2, SK-MEL-5, MaMel30I and MaMel27II were a generous gift from Boris Bastian at the University of California, San Francisco (UCSF); cell lines WM1366 (Cat N. WC00078), WM3629 (Cat N. WC00117), WM3670 (Cat N. WC00119) and WM3060 (Cat N. WC00126) were obtained from Coriell Institute (Wistar Institute, Philadelphia, PA, USA). The NRAS wild-type and BRAF mutated cell lines SK-MEL-28 and SK-MEL-5 were a generous gift from Boris Bastian. Cell lines DO4, MM415, MM485, SK-MEL-2, SK-MEL-28, SK-MEL-5 and MaMel30I were cultured in RPMI-1640 media supplemented with 10% (vol/vol) heat inactivated fetal bovine serum (FBS); cell lines WM1366, WM3629, WM3670 and WM3060 were cultured in MCDB153 media supplemented with 20% (vol/vol) Leibovitz's L-15 media, 2% (vol/vol) FBS, and 1.68 mM CaCl2. All cell lines were incubated at 37°C under 5% CO2.

### Drugs, cell viability assays, apoptotic assays, GI50 values

Palmostatin B was purchased from Merck Millipore (178501). ML348 and ML349 were purchased from Tocris Bioscience (USA). Cells were plated in 96-well plates with a density of 4000-8000 cells per well and incubated for 24 h at 37°C with 5% C02. Then cells were treated with increasing drug concentrations and their combinations. Cell viability was measured with the CellTiter-Glo Luminescent Cell Viability Assay (Promega; Wisconsin, USA) according to the manufacturer's protocol. Luminescence was measured on the SynergyHT plate reader (BioTek, Vermont, USA) using Gen5 software (Version 1.11.5). For apoptotic assays 0.1–0.2 × 10^6^ cells were plated in 12-well plates and treated with DMSO or an inhibitor. After 72 hours apoptosis was assessed using the Dead Cell Apoptosis Kit with Annexin V Alexa Fluor 488 and Propidium Iodide according to the manufacturer's instructions (Invitrogen, V13241) with the AccuriC6 Flow Cytometer using the CFlow software (Ver. 1.0.227.4). Concentrations of drugs resulting in 50% decrease in cell viability relative to DMSO treated controls (GI50) were calculated with CalcuSyn software (Biosoft, Cambridge, UK, Version 2.1).

### siRNA experiments

For siRNA studies cells were plated in 96 well plates (3–5 × 10^3^/well) or 60mm dishes (0.8 – 1.4 × 10^6^/dish). After 24 hours cells were transfected with SMARTpool siRNA directed against LYPLA1 or LYPLA2, both LYPLA1/LYPLA2, or the non-targeting control pool (all from Dharmacon, USA) at a final concentration of 50-100nM using Lipofectamine 2000 (Invitrogen, CA, USA) as per manufacturer's instructions. After 72 hours of incubation, cell viability was measured as described above, and 60mm dishes were subjected to cell lysis and immunoblots. The siRNA knockdown efficiency was quantified on the protein level and analyzed with the image processing software ImageJ (version 1.49d).

### Immunoblots

Cells were washed with phosphate buffered saline (PBS), lysed with radio-immunoprecipitation (RIPA) buffer [150 mM NaCl, 1% (vol/vol) Nonidet P-40, 0.5% (wt/vol) sodium deoxycholate, 0.1% (wt/vol) SDS] in 50mM Tris HCl (pH8.0) supplemented with protease and phosphatase inhibitors (Pierce, IL, USA; 78442). Protein concentrations were determined using the BCA Protein Assay kit (Pierce; 23225) according to the manufacturer's protocol. Proteins were separated by SDS-PAGE with 4-20% gradient gels (Bio-Rad Laboratories, CA, USA; 456-1096), transferred to Immobilon-P PVDF membranes (Millipore, MA, USA; IPVH00010), and blocked in 5% dry milk or 5% bovine serum albumin in Tris Buffered Saline, with Tween 20 (TBST) (Sigma-Aldrich). Membranes were incubated with primary and secondary antibodies, and target proteins were detected with ECL detection reagent (Pierce; 32106) or the SuperSignal West Dura substrate (Thermo Scientific, USA). β-Actin (Sigma-Aldrich) served as a loading control. Phospho-ERK (4370), ERK (4695), phospho-AKT (4060), AKT (4691), Phospho-S6 (4857) and S6 (2217) antibodies were obtained from Cell Signaling Technology (MA, USA). LYPLA1 (ab91600) and LYPLA2 (ab151578) antibodies were obtained from Abcam (MA, USA). The LYPLA1 and LYPLA2 knockdown efficiencies were analyzed using the software ImageJ (version 1.49d).

## SUPPLEMENTARY TABLE


