# Supplementary material for: Acyl protein thioesterase 1 and 2 (APT-1, APT-2) inhibitors palmostatin B, ML348 and ML349 have different effects on NRAS mutant melanoma cells
Source: Oncotarget. 2016 Jan 13;7(6):7297–306. doi: 10.18632/oncotarget.6907 (PMC4872786; doi:10.18632/oncotarget.6907)
Supplement: Supplementary file 1 [file oncotarget-07-7297-s001.pdf]

SUPPLEMENTARY TABLE

Supplementary Table S1: Growth inhibitory effects of palmostatin B in melanoma cell lines.

Melanoma Cell Line	Mutation	GI50 value [µM]
DO4	NRAS Q61L	77.53
MM415	NRAS Q61L	72.51
MM485	NRAS Q61R	>100
SK-MEL-2	NRAS Q61K	79.15
WM1366	NRAS Q61L	50.82
WM3060	NRAS Q61K	49.43
WM3629	NRAS G12D	27.98
WM3670	NRAS G12D	9.93
SK-MEL-28	BRAF V600E	>100

The table displays concentrations of drugs resulting in 50% decrease of cell viability relative to DMSO treated controls (GI50). Drug concentrations tested ranged from 1.5 to 100 µM.
